# Probabilistic single-particle cryo-EM *ab initio* 3D reconstruction in *SIMPLE*

**DOI:** 10.1107/S2059798325005686

**Published:** 2025-07-07

**Authors:** Cong T. S. Van, Cyril F. Reboul, Joseph J. E. Caesar, Rubén Meana-Pañeda, George T. Lountos, Justin C. Deme, Owain J. Bryant, Steven Johnson, Claire T. Piczak, Eugene Valkov, Susan M. Lea, Hans Elmlund

**Affiliations:** a National Cancer Institute National Institutes of Health Bethesda MD21701 USA; b Basic Science Program Frederick National Laboratory for Cancer Research Frederick MD21701 USA; c Structural Biology, St Jude Children’s Research Hospital, 262 Danny Thomas Place, Memphis, TN38105, USA; Rutherford Appleton Laboratory, United Kingdom

**Keywords:** cryo-EM, single particle, reconstruction, probabilistic, heterogeneity

## Abstract

Three-dimensional (3D) structure determination by single-particle analysis of cryo-electron microscopy (cryo-EM) images requires *ab initio* 3D reconstruction of density volume(s) from 2D images (particles). This large-scale inverse problem requires the determination of many million degrees of freedom from extremely noisy experimental measurements. Here, we introduce a new approach to probabilistic multi-volume *ab initio* 3D reconstruction for simultaneous estimation of the relative particle 3D orientations and partitioning of the particles into groups with distinct structural states. To account for further structural variability within the discrete state groups, due to for example regional disorder, flexibility or partial occupancy of associating ligands, we introduce a new method for adaptive non-uniform regularization based on iterated conditional modes (ICMs). Our ICM regularization approach can be viewed as a spatially varying real-space prior that optimizes the connectivity of the reconstructed density map(s). Our method is designed to run in real time as the microscope collects the data, which puts significant constraints on algorithm scalability and flexibility with regard to how new particles are incorporated. We describe the probabilistic optimization and non-uniform regularization theory in detail. Finally, we provide numerous benchmarking examples, both on publicly available standard test data sets and on data sets acquired at our cryo-EM facility at the National Cancer Institute, National Institutes of Health. The implementation of our new multi-volume *ab initio* 3D reconstruction approach is part of the *SIMPLE* software suite, which is provided open source at https://github.com/hael/SIMPLE.

## Abstract 2

We introduce a new approach to probabilistic multi-volume single-particle cryo-EM *ab initio* 3D reconstruction for simultaneous estimation of the relative particle 3D orientations and partitioning of the particles into groups with distinct structural states.

## 1. Introduction

All single-particle 3D reconstruction approaches, whether they are applied to *ab initio* analysis or orientation refinement, seek to identify the 3D volume(s) that best match the noisy experimental 2D images (particles). The relative 3D orientations of the particles are the latent variables of the model sought. Various methods have been applied to this large-scale inverse problem that involves many million degrees of freedom and extremely noisy experimental 2D measurements. The problem can be formulated in real space using the Radon transform (Radermacher, 1992 ▸, 1994 ▸; Lanzavecchia *et al.*, 1999 ▸) or in the Fourier domain using the projection slice theorem (Bracewell, 1956 ▸), which states that the Fourier transform of a 2D projection image of a 3D object is a central section through the origin of the Fourier transform of the 3D object. All 3D reconstruction approaches that are actively used in the field rely on the latter, Fourier-based, approach. Given a 3D reference Fourier volume, we can extract planes at known 3D orientations using convolution interpolation (Yang & Penczek, 2008 ▸; Penczek, 2010 ▸; Penczek *et al.*, 2004 ▸) and compare them with the Fourier-transformed particles. Convolution interpolation can also be applied to calculate a 3D reconstruction from particles with assigned 3D orientations (Penczek *et al.*, 2004 ▸; O’Sullivan, 1985 ▸; Beatty *et al.*, 2005 ▸; Jackson *et al.*, 1991 ▸). One way to identify maximum-likelihood estimates of parameters in statistical models depending on unobserved latent variables is through iterative fixed-point iteration algorithms, such as the expectation–maximization (EM) algorithm (Dempster *et al.*, 1977 ▸; Bishop, 2006 ▸). This involves updating the particle 3D orientations while keeping the current volume estimate fixed in the expectation step, followed by update of the 3D volume while keeping the particle 3D orientations fixed in the maximization step. Assuming that the estimation of the 3D orientation of each particle is independent of every other particle in the data set, this problem can be trivially decomposed into *N* independent subproblems, where *N* is the number of particles in the data set. This trivial decomposition method was implemented ∼20 years ago in the software package *FREALIGN* (Grigorieff, 2007 ▸), using a cross-correlation-based objective function for matching particles with volume re-projections and no statistical modeling of the interdependence between the latent variables associated with different particles.

The next leap in computational methods development for single-particle cryo-EM was the advent of the *RELION* program (Scheres, 2012*a* ▸), which utilizes an empirical Bayesian framework for 3D orientation estimation (Scheres, 2012*b* ▸). As is typical for maximum-likelihood (ML) methods (Sigworth, 1998 ▸), rather than assigning a single ‘optimal’ orientation for each particle, *RELION* calculates probability-weighted integrals over all possible orientations. Hence, each iteration in *RELION* involves associating each particle with a set of scalar weights mapping to a set of different 3D orientations, all of which are used for insertion of the 2D particle into the 3D Fourier volume being updated. *RELION* is based on regularized likelihood estimation in a discrete search space with a prior that limits the power of high frequencies in reciprocal space, imposing smoothness in a uniform manner in the real-space domain. Volume regularization based on estimation of the spectral powers of the signal and the noise is an elegant approach to reducing overfitting that is unique to *RELION*, and we refer to it as ML regularization here. However, just like *FREALIGN*, *RELION* assumes that the parameters controlling how the volume is updated for each particle are independent of every other particle in the data set.

Many attempts have been made to improve the inherently local orientation searches that are a consequence of designs based on trivial decomposition schemes (Joubert & Habeck, 2015 ▸; Jaitly *et al.*, 2010 ▸; Elmlund *et al.*, 2010 ▸, 2013 ▸; Elmlund & Elmlund, 2012 ▸; Reboul, Eager *et al.*, 2018 ▸; Vargas *et al.*, 2014 ▸; Singer *et al.*, 2010 ▸; Singer & Shkolnisky, 2011 ▸), but none have gained more popularity in practical applications than *cryo­SPARC* (Punjani *et al.*, 2017 ▸). The principal innovation in *cryoSPARC* was the first directly applicable solution to the single-particle 3D orientation search problem based on the establishment of global search directions in the parameter space, explored through stochastic gradient descent (SGD), a popular optimizer for large-scale machine-learning problems (Yang, 2024 ▸; Toader *et al.*, 2025 ▸). Like many other software packages, *cryoSPARC* relies on gridding interpolation of Cartesian 2D central sections from the 3D Fourier volume. In the *ab initio* 3D reconstruction step, these central sections are used to compute gradients of the likelihood with respect to the 3D density that are explored with SGD. Unfortunately, the *cryoSPARC* publications do not describe the computational methods in sufficient detail to allow re-implementation of their algorithms and the source code is closed. However, the *cryoSPARCab initio* 3D reconstruction approach is unique in two principal aspects. Firstly, the focus is on updating the volume rather than the 3D orientations of the particles. Hence, the 3D reconstruction step is an integral part of the method rather than an auxiliary step following the estimation of the latent variables that control how the volume is updated. Secondly, a global objective function is designed such that multiple particles can simultaneously influence the direction of the search. Letting all particles influence the search direction in each iteration would be intractable on any computer infrastructure, given the size of today’s data sets, which often contain many millions of particles. However, *cryoSPARC* uses the technique of importance sampling: a common approach to reducing the computational complexity of numerical integration both in terms of floating-point operations and memory usage (Maddouri *et al.*, 2022 ▸). This combination of stochastic sampling for computational efficiency with the estimation of global search directions explains the power of the *cryoSPARCab initio* 3D reconstruction approach, which can produce very high-quality maps directly from the noisy individual particles.

In this study, we asked whether global search directions could be obtained through coupling the ‘classic’ single-particle orientation search with probabilistic modeling. Like the earliest developed single-particle orientation search approaches (Penczek *et al.*, 1992 ▸, 1994 ▸; Frank *et al.*, 1996 ▸), our method is based on projection matching in polar coordinates, as described previously (Reboul, Kiesewetter *et al.*, 2018 ▸). Similarly to *RELION* and *cryoSPARC*, we use noise-weighted Euclidean distances to derive the probabilities that control how the particles are assigned rotational 3D orientations, while the rotational origin shifts are estimated through the continuous exploration of search directions defined by analytical gradients of the objective function. Our method explores a discrete space of rotational orientations, where each projection direction is represented by a polar reference 2D section, obtained through Fourier gridding interpolation from the Cartesian 3D Fourier volume. Rather than asking which 2D reference best matches a given particle, we construct a probability table over projection directions and in-plane rotations for all particles in the data set. Importantly, this table is updated following each individual particle 3D orientation assignment, thus allowing the decision made for one particle to influence the decisions made for all subsequent particles considered. We describe our method in sufficient mathematical detail to allow straightforward re-implementation in other packages and provide numerous benchmarking examples, both on publicly available standard test data sets and on data sets acquired at our cryo-EM facility at the National Cancer Institute (NCI), National Institutes of Health (NIH). The implementation of our new multi-volume *ab initio* 3D reconstruction approach is part of the *SIMPLE* software suite, which is provided open source at https://github.com/hael/SIMPLE.

## 2. Methods

### 2.1. Problem statement

Let {*P*_*i*_}, 1 ≤ *i* ≤ *N*_*P*_ be a set of 2D Cartesian central sections of the Fourier transform (FT) *V* of the real-valued 3D density *v* that is sought. These 2D projections or ‘particles’ are blurred by contrast transfer functions (CTFs) {*H*_*i*_} and have low signal-to-noise ratios (SNRs) due to the low-dose imaging deployed to prevent excessive radiation damage to the biological material. One way to attempt to solve the *ab initio* 3D reconstruction problem is to use a fixed-point iteration approach, *i.e.* starting from some initial *V*_0_, the FT of the initial density, reconstructed from {*P*_*i*_} at randomized orientations 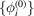, then finding another set of trial orientations 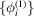 through some search procedure, and reconstructing a corresponding *V*_1_ from 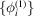. Fixed-point iteration approaches can be mathematically shown to guarantee convergence to a locally optimal estimate of *V*. We propose to combine fixed-point iterations with probabilistic assignment of the set of orientations 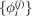 at each iteration *j*. Some notation: let ℘(*V*, ϕ) be the reprojection operator, taking the 2D central section of *V* in orientation ϕ, which contains a 3D rotation and a 2D vector describing the rotational origin shift, *i.e.* ϕ = (*o*, *s*), *o* ∈ *SO*(3), *s* ∈ *R*^2^. ||.|| denotes the Euclidean distance. In our approach, we discretize *SO*(3) to a uniform grid of size *N*_*m*_ × *N*_*n*_ over *S*^2^ × *S*^1^, where *N*_*m*_ is the number of references on *S*^2^ and *N*_*n*_ is the number of in-plane rotations on *S*^1^, so each *o*_*i*_ ∈ *SO*(3) corresponds to *o*_*m*,*n*_ ∈ *S*^2^ × *S*^1^. In the following paragraphs, we describe the building blocks of our method (objective function, orientation and particle sampling strategies, volume regularization *etc*.). Finally, we outline the overall algorithm design.

### 2.2. The objective function being optimized

An important consideration for any single-particle orientation search approach is the objective function being optimized. The most popular software packages in the field rely on Euclidean distance-based objective functions, where the distance contributions in the different resolution shells in reciprocal space are scaled based on the spectral powers estimated from the data (Scheres, 2012*a* ▸; Punjani *et al.*, 2017 ▸). We previously used a cross-correlation-based objective function in conjunction with matched filtering to reduce the effects of noise (Elmlund *et al.*, 2013 ▸; Reboul, Kiesewetter *et al.*, 2018 ▸; Caesar *et al.*, 2020 ▸; Elmlund & Elmlund, 2012 ▸; Reboul, Eager *et al.*, 2018 ▸; Reboul *et al.*, 2016 ▸). Cross-correlation-based approaches are attractive since they are independent of how the particles are normalized. However, we found that the spectral whitening associated with the matched filter often introduced overfitting when processing data with low SNR. To overcome this issue, we developed a Euclidean distance-based objective function, like that used in *RELION* for estimating orientation probabilities, but normalized to the [0, 1] interval to remove the dependency on image size and improve numerical stability. Let 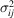 be the noise power of the *i*th particle *P*_*i*_, with orientation ϕ_*i*_ = (*o*_*i*_, *s*_*i*_), at Fourier index (resolution) *k*, estimated as
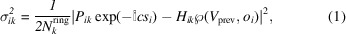
where 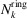 is the number of Fourier components in ring *k*, *c* is the phase-shift Jacobian constant and 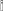 is the imaginary complex number. Assuming additive Gaussian noise, a Euclidean cost function of the *i*th particle with respect to some orientation ϕ = (*o*, *s*) can be formulated as
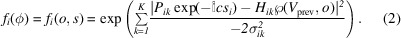
When the rotation *o*_*i*_ of the *i*th particle is known, the shift *s*_*i*_ can by found by a continuous optimization of the cost function, *i.e.*
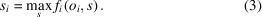
As previously described (Reboul, Kiesewetter *et al.*, 2018 ▸), we use the L-BFGS-B optimizer (Byrd *et al.*, 1995 ▸) with gradient with respect to the shift *s*
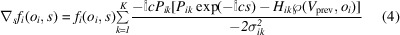
to estimate the origin shifts.

### 2.3. The angular threshold and the rotational sampling neighborhood

Let ε > 0 (in degrees) be a constant and let 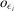 be the rotated version of *o*_*i*_ by a random rotation angle 0 ≤ ε_*i*_ ≤ ε; the Fourier volume reconstructed by the ε-bounded rotated orientations 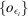 is then *V*_ε_, which is close to the truth *V* when ε is small. There is a threshold ε where *V*_ε_ starts to diverge significantly from *V*, and we call this threshold the angular threshold. For an angular threshold ε and an orientation *o*_*i*_, there is a physical neighborhood of orientations 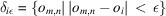 around *o*_*i*_ and a neighborhood of orientations γ_*i*ε_ = 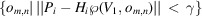 such that 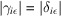 identified by evaluating some function that maps the elements of the search space to a scalar objective function value. If the objective function is perfect, we will have γ_*i*ε_ = δ_*i*ε_, *i.e.* the physical neighborhood matches the objective function-based neighborhood. We call this objective function-based neighborhood the sampling neighborhood, since we will use it to control the way that rotational orientations are sampled. The *SO*(3) sampling operator on γ_*i*ε_ is defined as 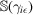. We also define the sampling operators over *S*^2^ and *S*^1^ as 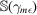 and 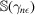, respectively. The sampling of rotational orientations is defined as follows.

(i) Identification of the space being searched as *S*^1^ (in-plane rotations) or *S*^2^ (projection directions).
(ii) Evaluation of the objective function values for the discretized points in the space considered.
(iii) Identification of an ε-neighborhood based on the current estimate of ε and calculation of a probability distribution through normalization within the neighborhood.
(iv) Stochastic sampling of the ε-neighborhood, just as a standard multinomial distribution would be sampled.

### 2.4. Probabilistic orientation assignment

The idea behind our probabilistic orientation assignment approach is to start with a sufficiently large sampling neighborhood when the estimate of *V* is random and adaptively adjust the sampling neighborhood in each iteration. In principle, the initial angular threshold could be set arbitrarily since it is updated as soon as a subset of particles have been assigned new 3D orientations. However, we found that an initial angular threshold of 10° provided an adequate convergence rate. In each iteration, each particle is probabilistically assigned a single projection direction, using the in-plane rotation sampled within the in-plane sampling neighborhood. Origin shifts are searched as described above.


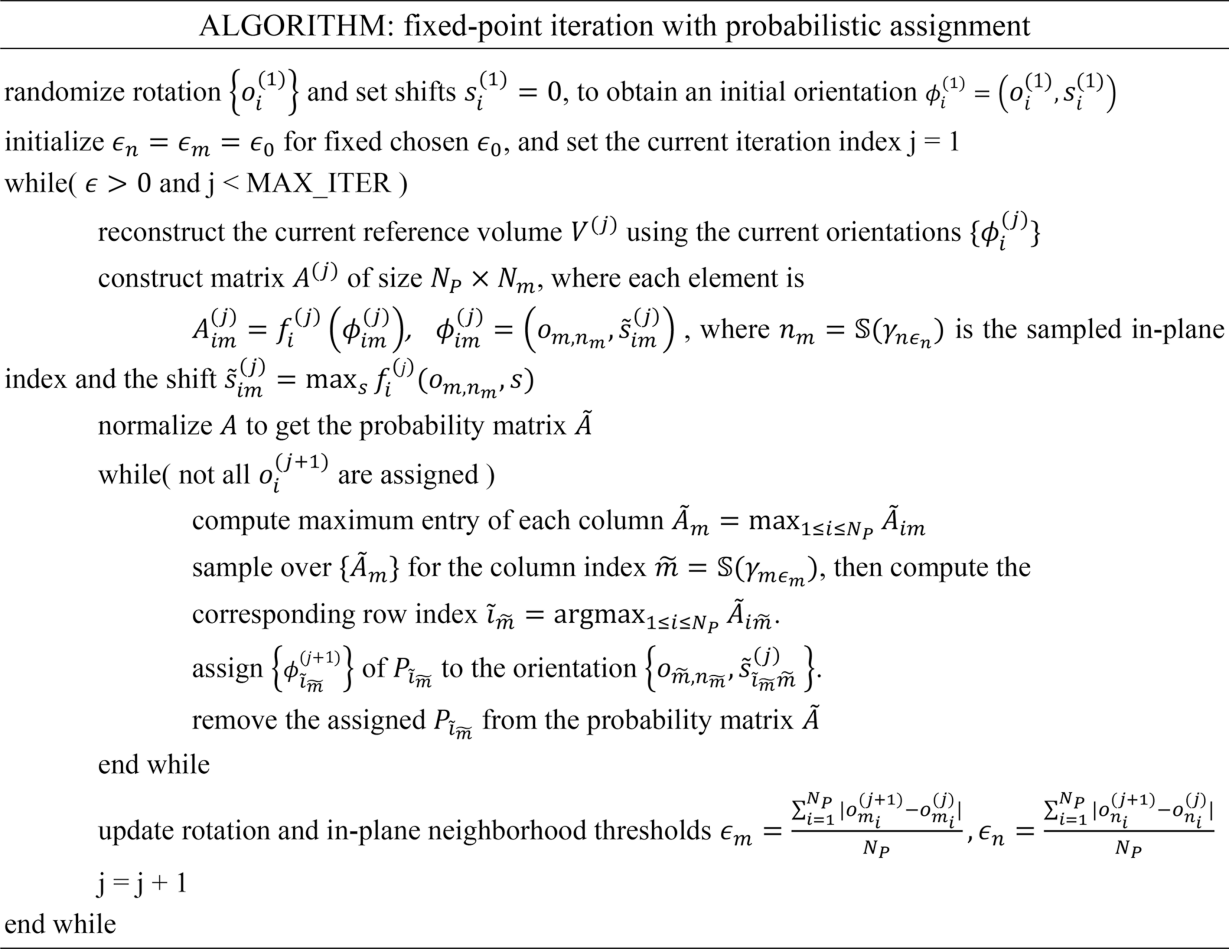


The normalization step to obtain the probability matrix 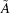 from *A* is as follows: let 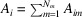 be the sum of each row of *A*, and normalize each row of *A* by its corresponding sum, *i.e.*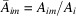, then perform min–max normalization of 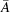 so that each matrix element has a value within the closed interval [0, 1].

### 2.5. A hybrid stochastic hill-climbing/probabilistic sampling approach for accelerated initial orientation search

We previously introduced stochastic hill-climbing (SHC) as an approach to increase the convergence radius of greedy approaches to single-particle 2D and 3D refinement (Elmlund *et al.*, 2013 ▸; Reboul, Kiesewetter *et al.*, 2018 ▸; Reboul, Eager *et al.*, 2018 ▸; Reboul *et al.*, 2016 ▸). Standard projection matching utilizes greedy hill-climbing, where the set of reference volume re-projections, either covering the entire asymmetric unit or representing a solid angle area in the vicinity of the previously assigned particle orientation, constitutes the search neighborhood. In greedy hill-climbing, the entire neighborhood is evaluated, and the particle is assigned the orientation of the best-matching re-projection. In contrast, SHC evaluates the projection direction neighborhood in random order and terminates the search once an improving orientation has been identified (first-improvement heuristic). This simple modification of the greedy approach to projection matching diversifies the search and increases the likelihood of convergence to a globally optimal solution. SHC does not require the generation of a large multi-dimensional matrix of probabilities, and due to the reduced number of re-projection comparisons its computational complexity is lower than that of standard projection matching. Therefore, we designed a hybrid search scheme for accelerating the orientation search in the early stages that combines probabilistic sampling of the in-plane rotations within the ε-neighborhood with SHC-based assignment of projection directions (see Section 2.9).

### 2.6. 2D class-restrained importance sampling

**Figure 1:**
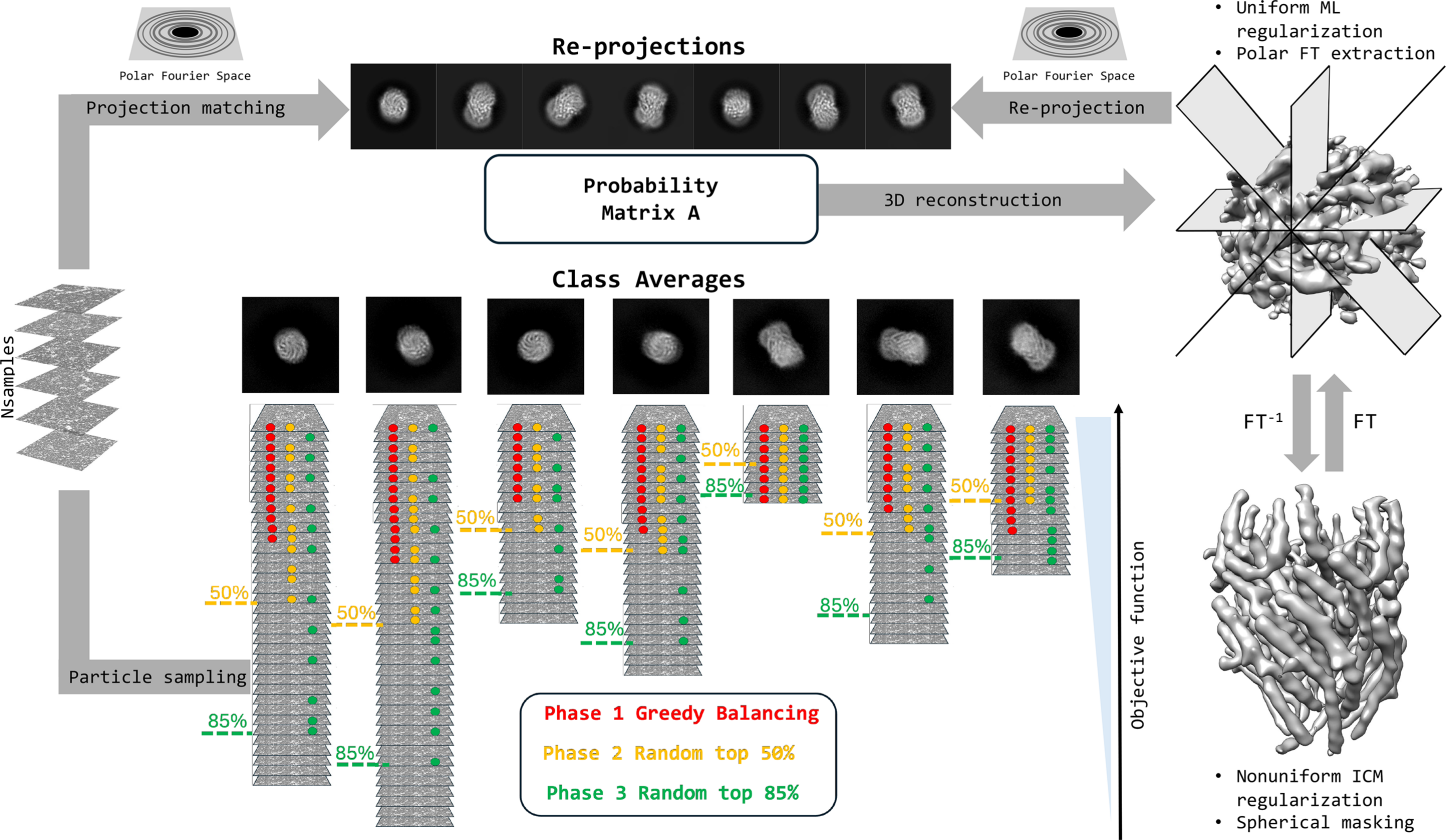
Schematic summary of our method for probabilistic *ab initio* 3D reconstruction. Particles are grouped into 2D classes prior to execution of the *ab initio* 3D workflow and signal-enhanced 2D class averages are calculated. Following random initialization of the volume, particles are sampled in a balanced fashion across the 2D classes. In phase 1, greedy balancing is performed, selecting the particles that best agree with their corresponding class average, as measured by the noise-normalized Euclidean distance. In phase 2, the particles that rank among the 50% most similar to the class average are sampled randomly. In phase 3, the fraction subjected to sampling is increased to 85%. The matrix of probabilities is obtained through matching the sampled particles with re-projections of the volume in polar Fourier space, allowing fast low-pass limited rotational matching. In each iteration, one element of the matrix describes the probability that a sampled particle was obtained from re-projection of the reconstructed volume in a certain projection direction and in-plane rotation, obtained through discretization of the *S*^2^ (2-sphere) and *S*^1^ (circle) manifolds, respectively.

Importance sampling in machine learning typically refers to preferential sampling of more important training examples that are likely to accelerate the training of the model sought. The computational complexity of our algorithm scales linearly with the number of particles. Hence, the selection of a subset of particles suitable for *ab initio* 3D analysis will reduce computations and potentially make the optimization problem more tractable. In *SIMPLE*, we have always relied on 2D analysis prior to *ab initio* 3D reconstruction for the selection of particles belonging to the class averages considered to be the best representations of the data set, both in terms of visual quality and objectively determined resolution (Caesar *et al.*, 2020 ▸; Elmlund & Elmlund, 2012 ▸; Reboul *et al.*, 2016 ▸). Here, we introduce a 2D class-restrained importance-sampling scheme for the selection of particles that are used to contribute to the estimation of the *ab initio* volume (see Fig. 1 ▸ for a schematic overview). The size of the subset of particles selected for inclusion is constant throughout the iterative optimization and is determined either automatically or by the user, whereas the sampling method changes automatically and dynamically as the optimization progresses. We deploy a balanced class sampling scheme, where particles are sampled evenly across the selected classes. In the first phase, we use a greedy balancing scheme that selects particles from classes such that the noise-normalized Euclidean distance function values from the 2D analysis are maximal. This ensures that high-contrast particles that agree well with the 2D class averages are used first, when the low-resolution shape of the molecule is being established. In the second phase, we randomly sample particles ranked among the top 50% evenly across the classes. In the third and final phase, we randomly sample particles ranked among the top 85% evenly across the classes. Thus, noisier particles that are more difficult to register are included later in the process, when the 3D reconstruction is of higher resolution, which increases the likelihood of producing a correct 3D registration of very noisy particles.

### 2.7. 2D class-driven frequency marching and downscaling coupled to automated low-pass limit estimation

Frequency marching refers to marching out radially in the Fourier domain from low to high frequency. This approach has been used for frequency-limited refinement to overcome noise-dependent overfitting since the beginnings of single-particle analysis (Grigorieff, 2007 ▸; Stewart & Grigorieff, 2004 ▸; Chen *et al.*, 2013 ▸) and it still plays a crucial role in modern single-particle analysis, especially in the steps of *ab initio* 3D reconstruction and multi-volume analysis for which proper twofold cross-validation (so-called gold-standard refinement; Scheres & Chen, 2012 ▸) is ineffective. In *SIMPLE*, twofold cross-validation for class resolution estimation and uniform regularization has been part of the 2D analysis since release 2.5 (Reboul *et al.*, 2016 ▸). In fact, a prerequisite for successful *ab initio* 3D reconstruction in *SIMPLE* is the generation of high-quality 2D class averages with projected secondary-structural elements clearly resolved. Each 2D class in *SIMPLE* has an associated twofold cross-validated Fourier ring correlation (FRC; Saxton & Baumeister, 1982 ▸) function from which the class resolution is estimated. To determine a suitable low-pass limit range for the 3D *ab initio* analysis, we select the starting low-pass limit to be the resolution at which the average FRC over all classes is 0.8 and the final low-pass limit to be the resolution at which the average FRC over the three best resolved classes is 0.143. The latter limit is restricted to the closed interval [4.5 Å, 6.0 Å] but it can also be set to lower resolution by the user. Next, we divide the entire *ab initio* 3D process into eight stages and define the low-pass limits of the six in-between stages by stepping linearly along the values of the average FRC over all classes. The downscaling factor applied in each stage is selected such that the sampling distance of the resulting particles matches one third of the low-pass limit estimated for that stage. Hence, in the initial stages of the search computations are substantially reduced through aggressive downscaling, while in the later stages the reduced downscaling allows reconstruction of the 3D density at sufficiently high resolution. The low-pass limits and downscaling factors estimated by this approach can be applied to successfully reconstruct most of the data sets that we have analyzed so far. However, for smaller particles with lower SNRs, the low-pass limits estimated by this approach do not accurately reflect the true resolution of the reconstructed 3D density. Therefore, we implemented an automatic low-pass limit estimation procedure that resembles how the non-uniform filter is estimated in *cryoSPARC* (Punjani *et al.*, 2020 ▸). Rather than minimizing the Euclidean distance between the even and the odd maps with respect to the resolution of a Butterworth filter function per voxel value, we minimize the sum of Euclidean voxel distances across the entire spherical 3D mask to obtain a uniform low-pass limit. Hence, the frequency limit that we use in the last two stages of the search is allowed to move dynamically between individual optimization iterations. We found that the final low-pass limits estimated by this approach typically lie between FSC = 0.5 and FSC = 0.143 (see Supplementary Fig. S2). In principle, this approach could constitute the basis for an alternative metric for resolution estimation, but this would require further investigation and benchmarking on well characterized data sets.

### 2.8. Non-uniform regularization by optimizing map connectivity in real space

Our approach uses uniform volume regularization to reduce overfitting and ensure real-space volume smoothness through the same ML regularization scheme as implemented in *RELION* (Scheres, 2012*a* ▸), *i.e.*
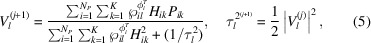
where 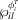 interpolates *P*_*i*_ (and *H*_*i*_) at the current orientation ϕ_*i*_ into the 3D Fourier grid at 3D Fourier index *l*. In addition, we introduce a new method for non-uniform (local) regularization. Punjani and coworkers put forward a general framework for optimization of the hyperparameters controlling the degree of smoothing introduced by regularization or filtering techniques (Punjani *et al.*, 2020 ▸). This non-uniform regularization approach, when coupled to the twofold cross-validated 3D refinement in *cryoSPARC*, provided adaptive regularization, thus addressing the issue that single-particle 3D refinement methods tend to simultaneously overfit and underfit data sets with significant variations in local resolution due to flexibility or the presence of disordered regions or detergent micelles. This approach can be summarized as follows.

(i) Create low-pass filtered representations of the even map using some uniform impulse-response function (cosine, Butterworth *etc.*).
(ii) Identify which filtered even map minimizes the Euclidean distance between each voxel and the corresponding voxel in the odd (raw) map.
(iii) Generate a non-uniformly filtered map by selecting the combination of optimally filtered voxels.

This approach recognizes that non-uniform regularization is inherently a real-space optimization problem and it has proven to be superior to uniform regularization approaches in single-particle 3D refinement. Here, we introduce an alternative method for non-uniform volume regularization based on iterated conditional modes (ICMs; Taylor, 2011 ▸; Pungpapong *et al.*, 2015 ▸) for optimization of map connectivity in real space. A Gibbs random field describes the statistical properties of an interconnected network of non-negative items (set of voxels). Our scenario is stationary in time and restricted to spatial neighborhood dependencies, *i.e.* voxel connectivity (the way in which pixels in three-dimensional images relate to their neighbors). ICM is a deterministic algorithm for obtaining a configuration of a local maximum of the joint probability of a Gibbs random field by iteratively maximizing the probability of each variable (voxel) conditioned on the others in the neighborhood. We obtain a noise volume through subtraction of the even map from the odd map, followed by estimation of per-voxel noise standard deviations σ_*i*_ through voxel neighborhood analysis. We then apply ICM for non-uniform volume regularization of the even and odd maps independently as follows


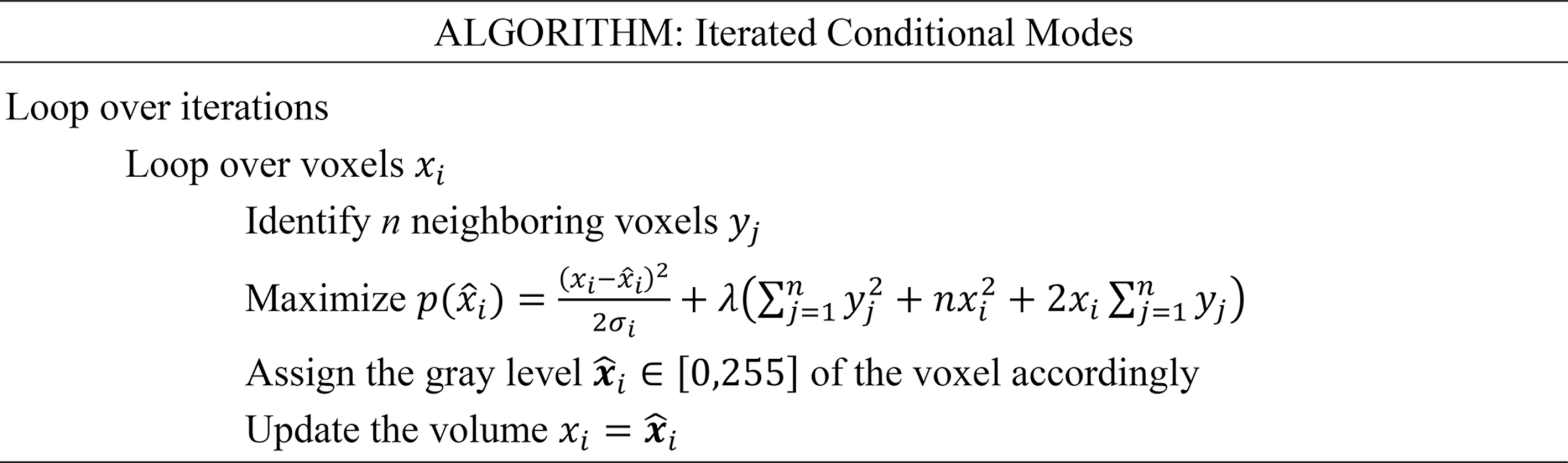
where λ = 1 is a regularization parameter. The discretization of the voxel values of the even and odd maps required for optimizing the local neighborhood quadratic potential is performed through vector quantization.

### 2.9. Overall algorithm design

Table 1 ▸ summarizes the overall algorithm design. The search is divided into eight stages. The degree of downsampling decreases with increasing stage number, and in each of the last 45 optimization iterations in stages 7–8 a low-pass limit is automatically estimated from the even/odd pair, as described above. The initial 3D orientations subjected to optimization can either be generated randomly or estimated through prior 3D registration of the class averages, followed by mapping of the identified class 3D orientations to the particles. We have found the latter initialization procedure to be the most effective in most cases. Following initialization, the hybrid SHC/probabilistic sampling approach described above is applied to search orientations in the first four stages. Once a low-resolution *ab initio* 3D density has been estimated in this way, the global probabilistic search is deployed. In the first two stages, no regularization is applied and strictly frequency-limited 3D registration is performed without applying any filter to the reference volume. In the following two stages, ML regularization is applied to denoise the reference volume. The ML regularization relies on estimation of the SSNR from the FSC, and a reliable FSC plot is not typically produced until stage 3 of the search. Once the global probabilistic search is switched on, we change regularization from uniform ML to non-uniform ICM, which is critically important for smaller membrane-protein structures but has comparably little influence on soluble proteins, which are typically composed of more ordered density due to the absence of a detergent micelle. Balanced particle selection over classes is performed greedily for the first three stages, selecting the top-ranking particles in each class, and then including noisier data as the search progresses (top-ranking 50% in stages 4–6 and top-ranking 85% in stages 7–8). The maximum number of projection directions used for matching is progressively increased from 500 (stages 1–2) to 1000 (stages 3–6) and 2500 (stages 7–8).

### 2.10. Generalization of the algorithm to multi-volume *ab initio* 3D reconstruction

Multi-volume analysis is frequently used in the *ab initio* 3D reconstruction step, either to distinguish between groups of particles with high versus low quality or to distinguish between groups of particles with different conformational or compositional states. The generalization of our algorithm to multi-volume *ab initio* 3D reconstruction is straightforward. When initial orientations are assigned, either randomly or through 3D registration of the 2D class averages, we simultaneously partition the particles randomly into different structural state groups. This corresponds to appending a set of 3D reference re-projections in the probabilistic search to account for the structural state labeling. In the terms of the matrix formulation of the global probabilistic search, we can think of an additional state as increasing the number of projection directions used to generate the matrix. Hence, increasing the number of states to extract by the procedure has the same effect on computational complexity as increasing the number of projection directions. We have also implemented a mode for multi-volume *ab initio* analysis where the state partitioning is performed later, just before the final stage of global probabilistic search. This mode assumes that the nature of the heterogeneity is such that it is meaningful to register the re-projections of the different co-existing structural states to one average volume at the beginning of the search. Further investigations into how to objectively determine the number of states to separate are ongoing, but our publicly available code currently supports multi-volume *ab initio* 3D reconstruction from random initialization at the start (het_mode=independent) or at the final stage (het_mode=docked). We provide a couple of examples demonstrating these utilities below.

### 2.11. Ensemble *ab initio* 3D volume analysis

Below, we describe numerous repeated benchmarking runs of our method on a variety of single-particle data sets. To simplify the presentation of our results and to avoid having to show all the reconstructed density maps obtained from a given data set, we implemented a tool for analysis of an ensemble of 3D volumes. The 3D orientation of an *ab initio* 3D map, as well as its absolute hand (Rosenthal & Henderson, 2003 ▸), is arbitrary. Our method for 3D registration of arbitrarily oriented 3D density maps with unknown handedness relies on pairwise *ab initio* docking of all possible pairs of inputted volumes in their two respective hands, followed by the calculation of a correlation-based metric of similarity stored in a matrix. Next, we analyze all pairwise correlation-based scores to determine which of the 3D volumes shows the best agreement with all of the other ones. The identified medoid volume is the representative volume of the ensemble: the one with the largest sum of similarities to all other members. No averaging of volumes is performed. Instead, the volumes of the ensemble are compared with the medoid volume, and possible outliers are identified through analysis of the correlation-based scores. We recognize that this kind of approach could be used to analyze heterogeneous ensembles of volumes, for example generated by *k*-fold cross-validation and/or multi-volume analysis repeated while varying the number of structural state groups to partition the data into. These kinds of analyses may become important when processing data sets for which consistent results are difficult to obtain upon repeated runs of *ab initio* 3D reconstruction.

## 3. Results

We previously introduced *SIMPLE* 3.0 for streaming single-particle analysis in real time (Caesar *et al.*, 2020 ▸), which focused on real-time data processing using minimal CPU computing resources to allow easy and cost-efficient scaling of processing as data rates escalate. Our streaming single-particle analysis tool in *SIMPLE* implements the steps of anisotropic motion correction and CTF estimation, rapid particle identification, extraction and 2D clustering. Below, we refer to the data sets processed by this approach, without excluding any particles after 2D analysis, as ‘streamed data sets’. We refer to data sets that only contain particles that have been identified as usable by previous 2D analysis as ‘cleaned data sets’. The data sets available for download at EMPIAR are typically cleaned data sets. Throughout the iterative process, the 3D maps produced by the *SIMPLE* multi-volume *ab initio* method are masked only with a soft-edged spherical mask, and no *B*-factor sharpening (Rosenthal & Henderson, 2003 ▸) is performed at any stage.

### 3.1. Single-volume *ab initio* 3D reconstruction from cleaned data sets

**Figure 2:**
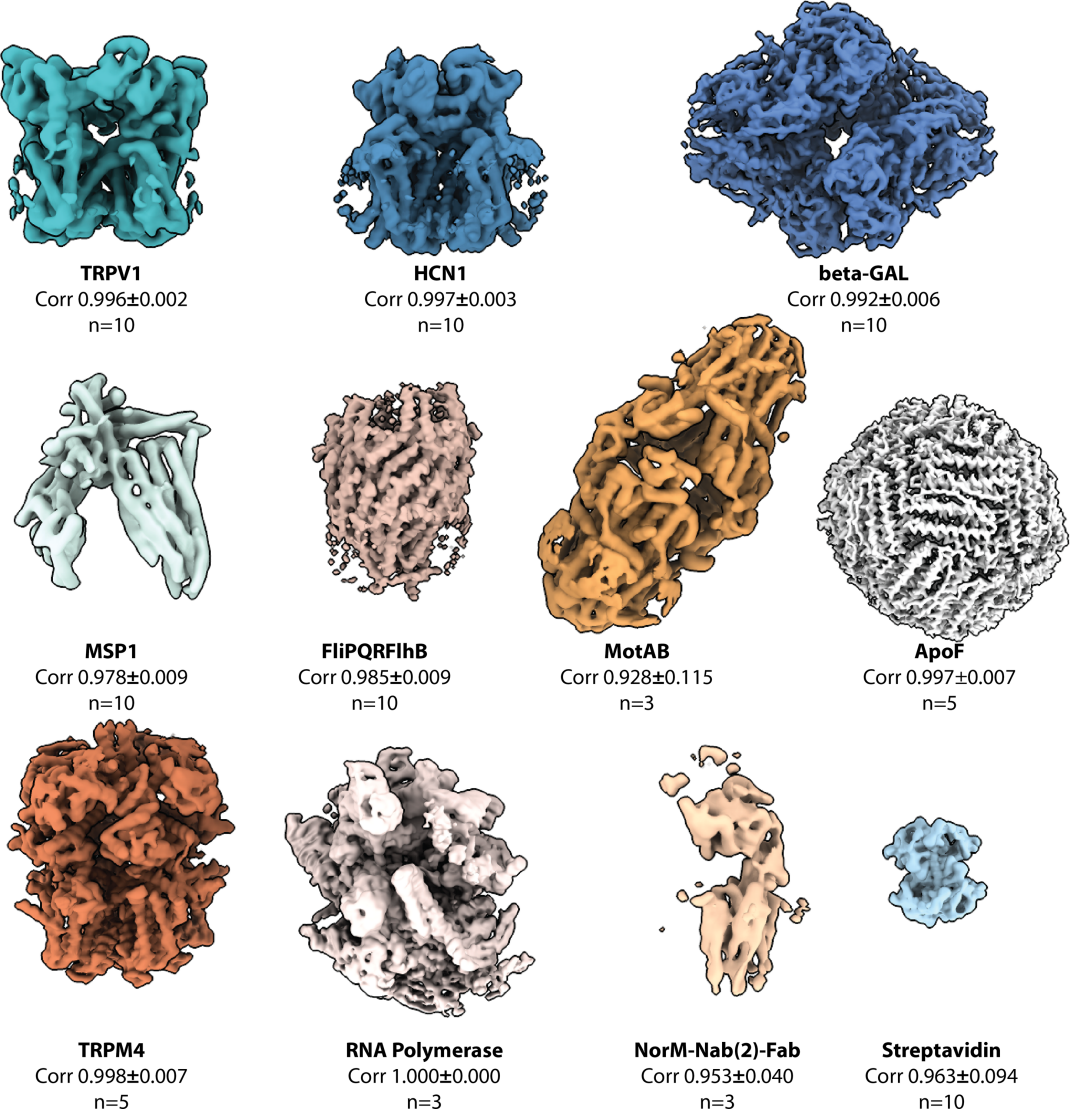
Single-volume *ab initio* 3D reconstructions obtained with the probabilistic approach implemented in *SIMPLE*. The number of independent repeats (*n*) is indicated together with the average correlation to the medoid of the ensemble, calculated to a resolution of 6 Å, and its standard deviation. Blue tone colors are used for volumes from cleaned data sets and orange/white tones for volumes from streamed data sets.

**Figure 3:**
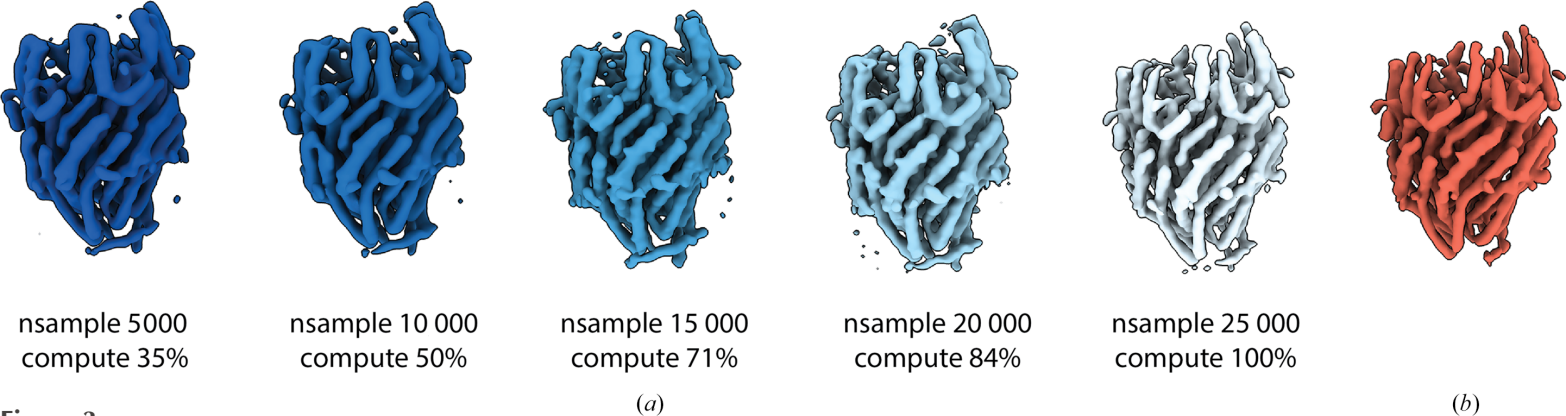
Impact of nsample on volume quality and compute time for the FliPQRFlhB complex. (*a*) Representative *ab initio* volumes calculated in *SIMPLE* using nsample values between 5000 and 25 000 with the computational cost relative to the time taken to calculate the 25 000 nsample volume below. (*b*) *Ab initio* volume obtained from the same particle set in *cryoSPARC*.

**Figure 4:**
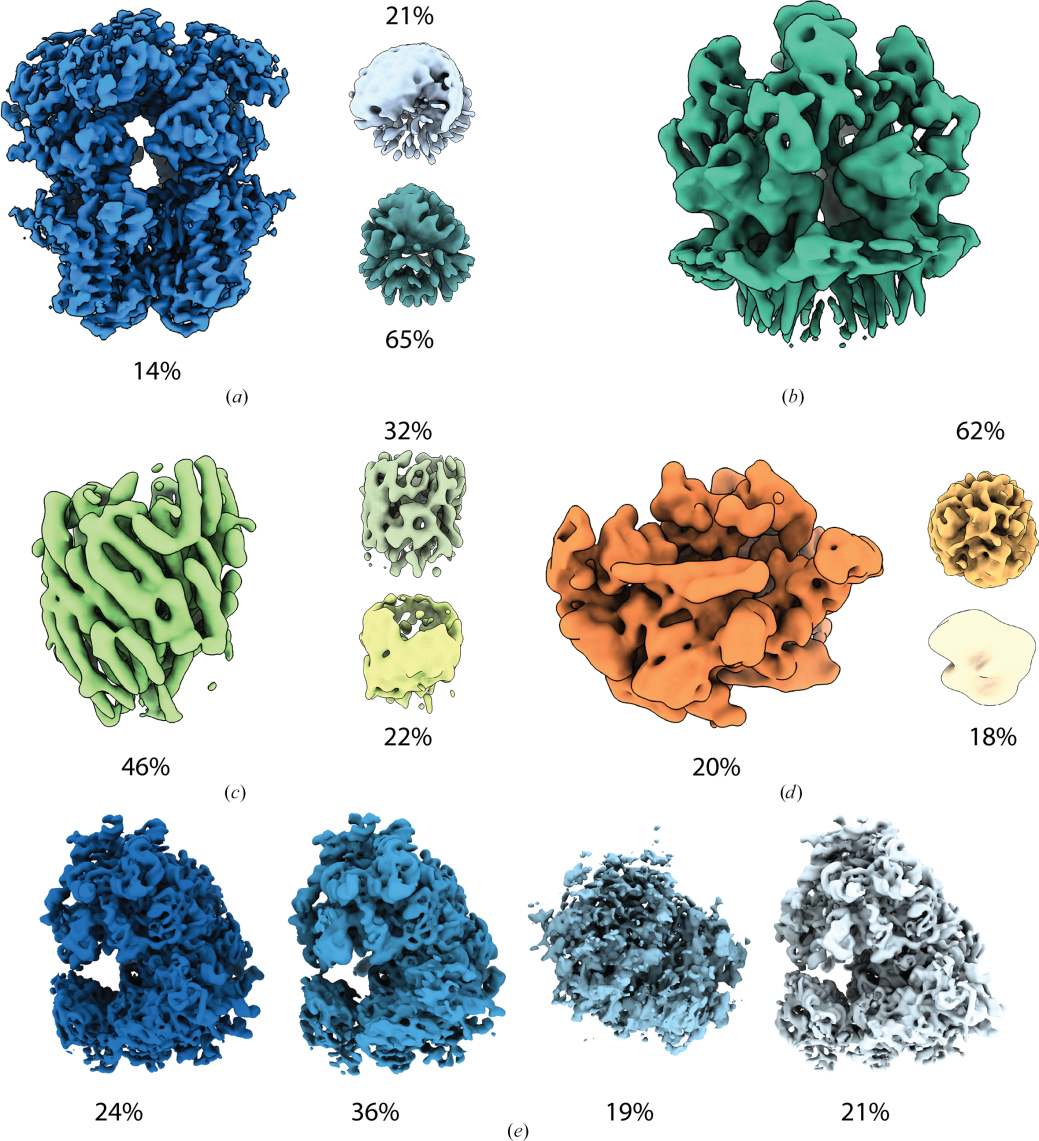
Finding useable particles in streamed data sets using *SIMPLEab initio*. For each sample, *SIMPLEab initio* was run directly on all particles from the stream without any manual or automatic 2D class rejections. The percentages report the distribution of particles between the different states. Three states were arbitrarily chosen for (*a*) TRPM4, (*c*) FliPQRFLhB and (*d*) RNA polymerase, whereas in (*e*) the ribosome particles were arbitrarily divided into four states. (*b*) shows a TRPM4 *ab initio* 3D reconstruction obtained from a subset of particles from the initial junk partition of 65%, identified by subjecting this partition to further 2D analysis, selection of 2D classes and single-state *ab initio* 3D reconstruction. This TRPM4 conformation is likely to correspond to a previously identified calcium-bound cold state (Hu *et al.*, 2024 ▸).

**Figure 5:**
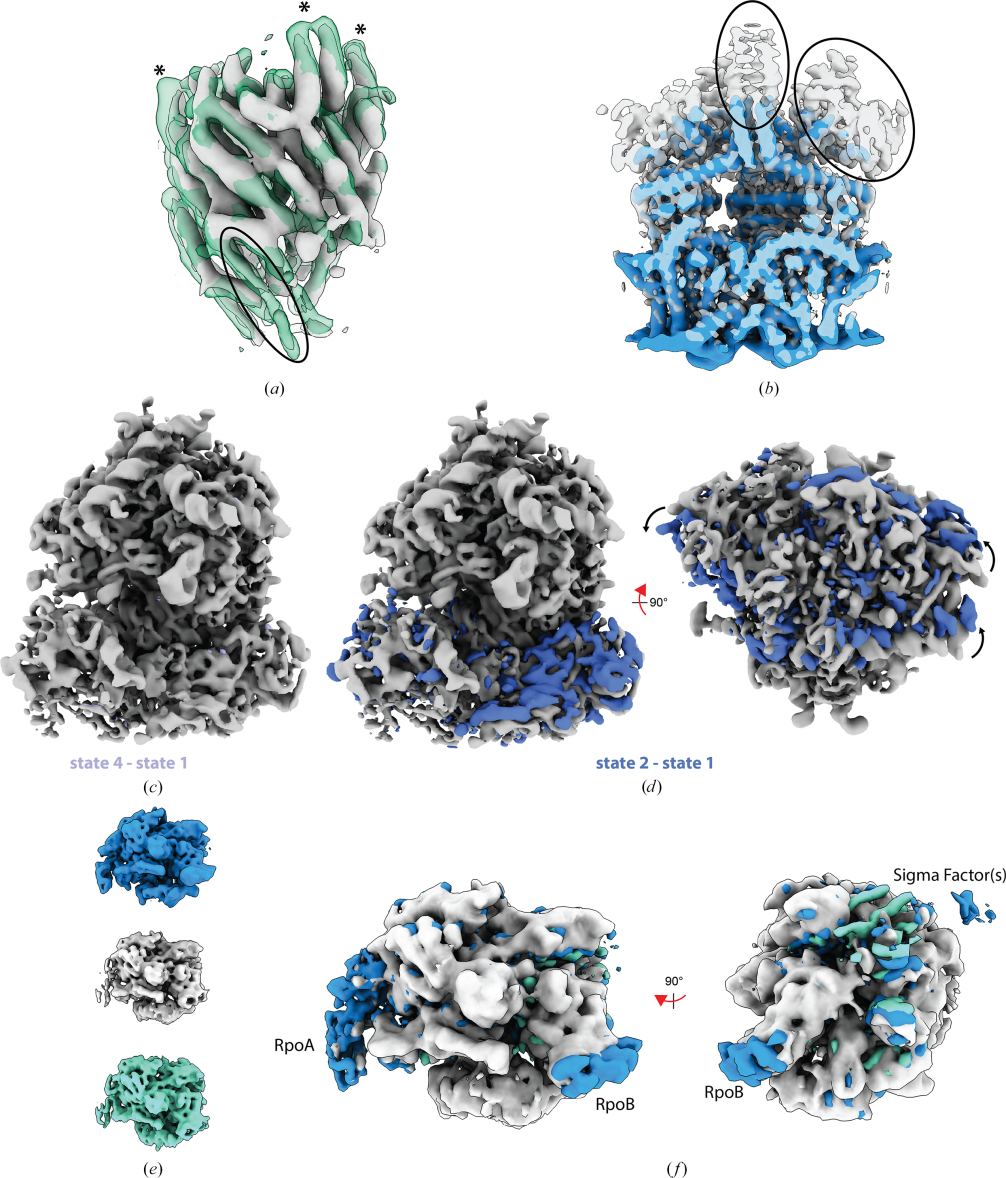
Separating conformational/compositional states in *SIMPLEab initio*. (*a*) Starting with the cleaned FliPQRFlhB data set and reconstructing two volumes (green semi-transparent surface and silver solid surface) separates particles with lower occupancy of the FlhB component (highlighted with a black ellipse) and less order in the intra-helix loops at the top (highlighted with asterisks). (*b*) Multi-volume reconstruction of TRPM4 separates particles in which the N-terminal domains and C-terminal coiled coil are well ordered (silver, semi-transparent surface) and where these are not well ordered (blue surface). (*c*) and (*d*) compare the three ‘good’ ribosome reconstructions obtained directly from the stream (see Fig. 3 ▸) and reveals that (*c*) states 1 and 4 are conformationally consistent (state 1 shown as a silver surface, with difference density calculated in *ChimeraX* overlaid as a lilac surface) and that (*d*) states 2 and 1 differ in the orientation of the small ribosomal subunit with respect to the large ribosomal subunit (state 1 shown as a silver surface, with difference density calculated in *ChimeraX* overlaid as a blue surface). The arrows indicate the major movement of the small ribosomal subunit between the two volumes overlaid on the large ribosomal subunit. (*e*) Three states generated by multi-volume *ab initio* 3D reconstruction on cleaned RNA polymerase data. (*f*) One state is shown in silver with the difference density for the other two states shown colored as in (*e*).

We first wanted to validate the capability of our algorithm to identify approximate relative 3D orientations of the particles and reconstruct initial *ab initio* volumes amenable to high-resolution 3D refinement. In the first phase of testing, we processed cleaned data sets available for download at EMPIAR. Our independent *ab initio* 3D reconstruction runs were repeated ten times for each of the data sets (see Fig. 2 ▸) of the *D*2-symmetric 0.5 MDa β-galactosidase (5000 particles selected from EMPIAR-10012), the *C*4-symmetric 400 kDa TRPV1 ion channel (EMPIAR-10005), the *C*4-symmetric 320 kDa human HCN1 hyperpolarization-activated cyclic nucleotide-gated ion channel (EMPIAR-10081), the main conformation of the asymmetric 225 kDa full-length merozoite surface protein 1 from *Plasmodium falciparum* (EMPIAR-10437) and the *D*2-symmetric 52 kDa streptavidin (EMPIAR-10335). Only one of the 50 runs on cleaned data sets was unsuccessful. The one outlier *ab initio* 3D volume detected by our medoid ensemble volume analysis represented a failed run for the 52 kDa streptavidin. We next turned to a data set of the flagellar export gate (Kuhlen *et al.*, 2020 ▸) that was not possible to reconstruct using the traditional approach of generating an *ab initio* 3D volume from 2D class averages and using that to initialize 3D refinement of the individual particles, as implemented in previous versions of *SIMPLE* (Reboul, Eager *et al.*, 2018 ▸, Reboul *et al.*, 2016 ▸), but had been successfully reconstructed using the SGD approach in *cryoSPARC* (Punjani *et al.*, 2017 ▸). We obtained 10/10 successful *ab initio* 3D reconstructions of the export gate (labeled FlipQRFlhB in Figs. 2–5 ▸ ▸ ▸ ▸) from particles identified as usable by the *SIMPLE* 2D analysis (Caesar *et al.*, 2020 ▸). Supplementary Fig. S1 shows the medoid volumes with fitted atomic coordinates for all of the cleaned data sets analyzed. Supplementary Fig. S2 shows all of the FSC plots and compares the final low-pass limit estimated by our approach with the resolution limits at FSC = 0.5 and FSC = 0.143, respectively.

### 3.2. Robustness of the 2D class-restrained importance sampling

Next, we wanted to assess the robustness of the 2D class-restrained importance sampling scheme (schematic overview in Fig. 1 ▸) to the number of particles sampled. To this end, we ran independent *ab initio* 3D reconstruction runs for the export gate data set while decreasing the number nsample of balanced particle samples from 25 000 to 5000 (see Fig. 3 ▸). The export gate could be reliably reconstructed from as few as 5000 samples, but further decreasing the number of samples was not possible. The implication of this finding is important for the future integration of our probabilistic *ab initio* 3D method into the *SIMPLE* real-time streaming pipeline, as it shows that as soon as good class averages have been obtained in the stream, high-quality *ab initio* 3D reconstructions can be obtained rapidly using class-balanced sampling. Conceivably, the class-balanced sampling strategy could be coupled to the state of the stream in terms of number of particles harvested and their statistical characteristics identified by the real-time 2D analysis.

### 3.3. Single-volume *ab initio* 3D reconstruction from streamed data sets

Single-state *ab initio* volumes were generated from particles selected from streaming data processing at the 2D class-average level. The cryo-EM data-collection and biological sample information is described in Supplementary Table S1. Several independent *ab initio* repeats were run for each data set, using class averages to provide initial estimates of the particle 3D orientations. The volumes were docked and correlations between repeats were calculated using the ensemble volume analyzer with the number of repeats, mean and standard deviation of the correlation to the medoid reported in Fig. 2 ▸. These results highlight the reliability and robustness of our *ab initio* approach in generating interpretable 3D maps from data sets subjected to minimal stream processing with our automated pipeline at a stage when only crude 2D analysis and no prior 3D analysis has been performed.

### 3.4. Multi-volume *ab initio* 3D reconstruction to identify usable particles from streamed data sets

We validated the multi-volume *ab initio* 3D reconstruction approach on simulated data (see supporting information). Next, we wanted to test whether we could identify subsets of particles in large data sets that would average well together to give interpretable structural information. Although the user can select which 2D classes to use for downstream processing or deploy unsupervised ‘good’ 2D class identification in *SIMPLE*, there is a potent risk that rare views are omitted or that user bias is introduced by these approaches. Therefore, we wanted to assess whether we could sort the particles in 3D without any manual intervention or automatic 2D class rejection. To this end, we arbitrarily executed *ab initio* runs in three states for several samples with no selection of particles at the 2D class-average level. In each case, interpretable *ab initio* volumes were generated (see Fig. 4 ▸), even when the fraction of useable particles was as low as 14% (see Fig. 4 ▸*a*, TRPM4). Furthermore, *SIMPLE* can separate particles into partitions with ordered versus unordered structural regions (see Figs. 5 ▸*a* and 5 ▸*b*) as well as identifying partitions of particles with distinct conformational states, as demonstrated by our analysis of a data set of the human ribosome, where partitions of particles with the small ribosomal subunit rotated with respect to the large ribosomal subunit could be distinguished from a partition of particles that did not generate any interpretable structural information (Figs. 4 ▸*e*, 5 ▸*c* and 5 ▸*d*). For each data set, the particles associated with each state group were analyzed by 2D classification. The particles associated with the states with interpretable 3D maps yielded high-resolution class averages, while class averages generated from particles associated with the other (junk) states were either not the biological sample or of much lower resolution. For TRPM4, the particles associated with the large junk-state group (65%) gave class averages that were a mixture of contaminations/junk (79%) and a lower resolution, yet distinct, conformational state of TRPM4. Using these particles (21% of the original state group, 14% of the total particle set) in a single-state *ab initio* 3D run generated an interpretable volume (see Fig. 4 ▸*b*). Our best-resolved TRPM4 structural state (see Fig. 4 ▸*a*) is conformationally similar to the structure determined by Autzen *et al.* (2018 ▸), while the other conformation (see Fig. 4 ▸*b*) reflects the less well packed, expanded form of TRPM4 previously identified as a calcium-bound cold state by Hu *et al.* (2024 ▸). The difference in stability of the two states is likely to explain the difference in resolution of the volumes, despite similar final particle numbers being assigned to each state group.

### 3.5. Multi-volume *ab initio* 3D reconstruction to identify co-existing structural states in cleaned data sets

Starting with the cleaned export gate (FliPQRFlhB) data set and reconstructing two volumes separates particles with lower occupancy of the FlhB component and less order in the intra-helix loops at the top (see Fig. 5 ▸*a*). Multi-volume reconstruction of TRPM4 from a cleaned data set separates particles in which the N-terminal domains and C-terminal coiled coil are well ordered versus not well ordered (see Fig. 5 ▸*b*). A cleaned subset of particles for the bacterial RNA polymerase was used in a three-state multi-volume *ab initio* run and resulted in the volumes shown in Fig. 5 ▸(*e*) with roughly a third of the particles in each state. Overlaying the volumes with coordinates for the polymerase complex (PDB entry 8reb) suggest that the three states differ in the ordering/orientation of the RpoA thumb region, in the second and third domains of RpoB and in the presence or absence of bound sigma factors (see Fig. 5 ▸*f*).

## 4. Discussion

The probabilistic *ab initio* 3D reconstruction approach that we have developed in *SIMPLE* lies somewhere between the 3D refinement approach implemented in *RELION* and the *ab initio* 3D reconstruction algorithm of *cryoSPARC* (see Table 2 ▸), but it also relies on concepts that were part of the original projection-matching implementations (Penczek *et al.*, 1992 ▸; Hohn *et al.*, 2007 ▸; Frank *et al.*, 1996 ▸; Grigorieff, 2007 ▸; Stewart & Grigorieff, 2004 ▸; Ludtke *et al.*, 1999 ▸). Similarly to *RELION* and *cryoSPARC*, we use noise-normalized Euclidean distances to guide the orientation search. However, our implementation is unique in that we formulate the orientation search in polar coordinates. In contrast to *RELION*, which relies on iterative statistical estimation to overcome the need for optimization through weighted particle averaging, we use the objective function to control the probabilistic search decisions that assign one particle one orientation, rather than assigning one particle a distribution of orientations with weights. In this respect, our approach is more like *cryo­SPARC*. The uniform data-driven regularization originally implemented in *RELION* (ML regularization) is part of our method, but we also introduce a new way of performing non-uniform data-driven adaptive regularization through ICM.

2D multireference refinement has been an important diagnostic tool in single-particle analysis since *RELION* became widely used in the field (Zivanov *et al.*, 2019 ▸; Scheres *et al.*, 2005 ▸, 2012*a* ▸), and it is essential for validation of the quality of the data analyzed in real time using the *SIMPLE* streaming pipeline (Caesar *et al.*, 2020 ▸; Reboul, Eager *et al.*, 2018 ▸; Reboul *et al.*, 2016 ▸). Cryo-EM investigators often inspect the 2D class averages to determine whether a data set is worth pursuing for 3D structure determination. Earlier generations of single-particle analysis software packages relied heavily on the generation of 2D class averages for the calculation of initial *ab initio* 3D volumes subjected to 3D refinement (Penczek *et al.*, 1996 ▸; van Heel, 1987 ▸, 1989 ▸; Frank & van Heel, 1982 ▸; van Heel & Frank, 1981 ▸; Hohn *et al.*, 2007 ▸; Tang *et al.*, 2007 ▸; Grant *et al.*, 2018 ▸), but this approach went out of fashion with the introduction of the SGD optimization for *ab initio* 3D analysis in *cryoSPARC* (Punjani *et al.*, 2017 ▸). Here, we introduce a novel importance-sampling scheme that tightly couples the multireference 2D analysis with the *ab initio* 3D analysis through (i) prioritizing the sampling of particles with the highest similarity to selected class averages in the early stages in attempt to reduce bias due to noise and particle heterogeneity, (ii) balanced sampling across the 2D classes throughout all stages of the optimization in attempt to reduce bias due to preferred particle orientations and (iii) rapidly initializing the 3D particle orientation search using the class averages, similarly to traditional single-particle analysis approaches. In addition to letting the ensemble of 2D classes control particle sampling and provide the option for 3D orientation initialization, we use the resolution estimates of the 2D classes to control the degree of downsampling and estimate the low-pass limit bound at the various stages of optimization. Deriving constraints from the multireference 2D analysis to accelerate and make the subsequent *ab initio* 3D analysis more robust is unique to *SIMPLE* and we foresee that this is an area that will see further development in the future.

It has been argued that 3D reconstruction algorithms that require tuning of arbitrary parameters may lead to bias or at least subjectivity of the results (Scheres, 2012*b* ▸). However, any algorithm of this level of complexity will have tunable parameters, even if they are hidden from the user. In *cryoSPARC*, for example, selection of an appropriate size of the stochastic mini batches used in SGD can be critically important. Likewise, the low-pass limit bounds can in principle be manually adjusted by the user, although the default values typically work well. In the ML regularization of *RELION* there is a fudge factor (tau) that we keep at a constant value of 3 throughout the process (a higher value leads to less smoothening of the map). Our non-uniform ICM regularization requires a lambda regularization parameter that we keep at a constant value of 1, which works well for the purpose of *ab initio* 3D reconstruction, but using a constant lambda regularization term may have to be revisited when we redesign the method for high-resolution refinement. The most critical adjustable parameter for the user of our approach is *n*, which controls how many class-balanced particle samples are drawn in each iteration. Reducing nsample to a few thousand particles can lead to the production very high-quality *ab initio* 3D maps in a couple of hours on a typical CPU workstation, even for data sets of particles that would be considered challenging from a molecular-weight perspective, whereas other targets (typically small membrane-protein structures) require nsample to be ∼100 000 for successful *ab initio* 3D map generation, which would take a day or two of compute on a typical CPU workstation and would be better executed in a distributed CPU computing environment. *SIMPLE* supports both modes of execution. A reasonable restart strategy for a user processing single-particle data of an unknown structure with *SIMPLE* would be to try nsample = 5000 first, which works well for all of the data sets analyzed here, and increment nsample by 10 000 until a satisfactory *ab initio* volume is obtained. The ensemble of *ab initio* volumes obtained can be analyzed with our *volanalyze* tool to identify outliers and validate the results before pursuing high-resolution 3D refinement or more sophisticated structural heterogeneity analysis.

Many elegant multi-volume 3D reconstruction procedures have been proposed for partitioning the single-particle ensemble into discrete state groups with distinct structural characteristics (Scheres *et al.*, 2007 ▸; Gao *et al.*, 2004 ▸; Penczek *et al.*, 2011 ▸; Elmlund & Elmlund, 2012 ▸; Punjani *et al.*, 2017 ▸; Lyumkis *et al.*, 2013 ▸). These methods typically rely on maximum-likelihood estimation, treating the state assignments as hidden unobserved variables that are estimated with an expectation–maximization procedure. Distinguishing between particles that average well together to generate interpretable structural information from those that do not has become one of the main applications of multi-volume analyses. We had not anticipated that particles that cannot be averaged together to create high-resolution 3D structural information would cluster in any meaningful way amenable to separation by methods based on center-based clustering, which is the general statistical framework that most of these methods rely on. However, it has been found empirically in many studies that ‘good’ particles can be identified with approaches of this kind, albeit at a high computational cost and often involving extremely convoluted workflows. Multi-volume *ab initio* 3D reconstruction, as implemented in our current workflow, is by no means intended to constitute the definitive solution, either for the identification of which particles are usable or to model the structural heterogeneity of a cleaned data set. The importance-sampling scheme was designed to be optimal for *ab initio* 3D reconstruction in one state group, and more inclusive sampling schemes may have to be considered when refining the structural state partitioning. Nevertheless, our current scheme ought to be able to produce some initial insights into the nature of the structural heterogeneity in a data set and provide an initialization point for further refinement, and it works well for identifying groups of particles that can be used to generate high-quality 3D reconstructions in a streaming scenario. One drawback that it shares with all other approaches used in the field is that the number of states to separate must be inputted by the user. Another drawback is that the user must decide on some aspects of the nature of the heterogeneity (independent versus docked mode). Many of the image-processing ideas that we present warrant further investigation and comparative benchmarking versus other approaches. This will be addressed in our future studies.

## 5. Conclusions

The probabilistic framework for single-particle 3D orientation search introduced here will constitute the basis for our future efforts in completely automating the cryo-EM structure-determination process. Furthermore, it will be a critical component of the integrated platform for real-time cryo-EM structure determination developed by the *SIMPLE* team at the NCI/NIH. Our latest *SIMPLE* release, available for download at https://github.com/hael/SIMPLE, features an easy-to-use web-based graphical user interface (GUI) that can be run on any device (workstation, laptop, tablet or phone) and supports a remote multi-user environment over the network.

## Supplementary Material

Supplementary Table and Figures and validation of multi-volume ab initio 3D reconstruction on simulated data. DOI: 10.1107/S2059798325005686/ic5124sup1.pdf

## Figures and Tables

**Table 1:** Overall algorithm design

Stage	Orientation search	Regularization	Balanced particle selection	Shift search	Maximum No. of projection directions	Maximum No. of iterations
1	Hybrid SHC/probabilistic	None	Top ranking	No	500	20
2	Hybrid SHC/probabilistic	None	Top ranking	No	500	20
3	Hybrid SHC/probabilistic	ML	Top ranking	Yes	1000	17
4	Hybrid SHC/probabilistic	ML	Top 50%	Yes	1000	17
5	Probabilistic	ICM	Top 50%	Yes	1000	17
6	Probabilistic	ICM	Top 50%	Yes	1000	17
7	Probabilistic	ICM	Top 85%	Yes	2500	15
8	Probabilistic	ICM	Top 85%	Yes	2500	30

**Table 2:** Comparing our method with other approaches

	Projection matching (*EMAN*, *Spider*, *Sparx*)	*RELION*	*SIMPLE*	*cryoSPARC*
Type of algorithm	Iterative greedy	Iterative statistical estimation	Iterative probabilistic	Iterative probabilistic
Objective function	Cross-correlation	Noise-normalized Euclidean distance	Noise-normalized Euclidean distance	Noise-normalized Euclidean distance
Type of optimization	Local search	None	Probabilistic (one particle, one orientation)	Probabilistic
Search geometry	Polar	Cartesian discrete	Polar	Unknown
	Discrete	Discrete	Discrete in rotations	
			Continuous in shifts	
Regularization	FSC-based	ML regularization	ML regularization	Unknown
			ICM	
