# Supplementary material for: Probabilistic single-particle cryo-EM ab initio 3D reconstruction in SIMPLE
Source: Acta Crystallogr D Struct Biol. 2025 Jul 7;81(Pt 8):396–409. doi: 10.1107/S2059798325005686 (PMC12315586; doi:10.1107/S2059798325005686)
Supplement: Supplementary file 1 [file d-81-00396-sup1.pdf]

STRUCTURAL BIOLOGY

Volume 81 (2025)

Supporting information for article:

Probabilistic single-particle cryo-EM *ab initio* 3D reconstruction in *SIMPLE*

Cong T. S. Van, Cyril F. Reboul, Joseph J. E. Caesar, Rubén Meana-Pañeda, George T. Lountos, Justin C. Deme, Owain J. Bryant, Steven Johnson, Claire T. Piczak, Eugene Valkov, Susan M. Lea and Hans Elmlund

Table S1 Cryo-EM data collection and biological sample information

**Sample**	**EM**	**Detector**	**Pixel Size (Å)**	**Dose (e-/Å^2)**	**Movies**	**Picked Particles**	**Acknowledgements**
Endogenous *H. sapiens* 80S Ribosome from HEK293T	TitanKrios 300kV	Falcon 4i	1.464	54	20,165	759,963	Eugene Valkov<br>Claire Piczak
Endogenous *S. enterica* RNA Polymerase	Titan Krios 300kV	Falcon 4i	0.732	62.2	8,260	520,487	Owain Bryant
C.sporogenes MotAB-FliG domain fusion	Titan Krios 300kV	Falcon 4i	1.386	56.5	49,250	3,589,661	Johnson S *et al*. Structural basis of directional switching by the bacterial flagellum. Nat Microbiol. 2024 May;9(5):1282-1292.
Human Apoferritin	Titan Krios 300kV	Falcon 4i	0.693	51.8	6,025	1,872,069	Vitroease sample purchased from Thermofisher (cat #A51362)
V. mimicus FliPQR-FlhB complex	Titan Krios 300kV	Gatan K2	0.822	48	13,765	3,560,800	Kuhlen L *et al*. The substrate specificity switch FlhB assembles onto the export gate to regulate type three secretion. Nat Commun. 2020 Mar 10;11(1):1296.
NorM-Nab(2)-Fab complex	Titan Krios 300kV	Falcon 4i	1.464	52.4	7,680	1,736,180	Owain Bryant based on J.S. Bloch, S. *et al*. Development of a universal nanobody-binding Fab module for fiducial-assisted cryo-EM studies of membrane proteins, *Proc. Natl. Acad. Sci. U.S.A.* 118 (47) e2115435118, (2021).
H. sapiens TRPM4	Titan Krios 300kV	Falcon 4i	1.464	55	10,720	882,677	Owain Bryant

S1. Validation of single-volume *ab initio* 3D reconstruction from cleaned data sets

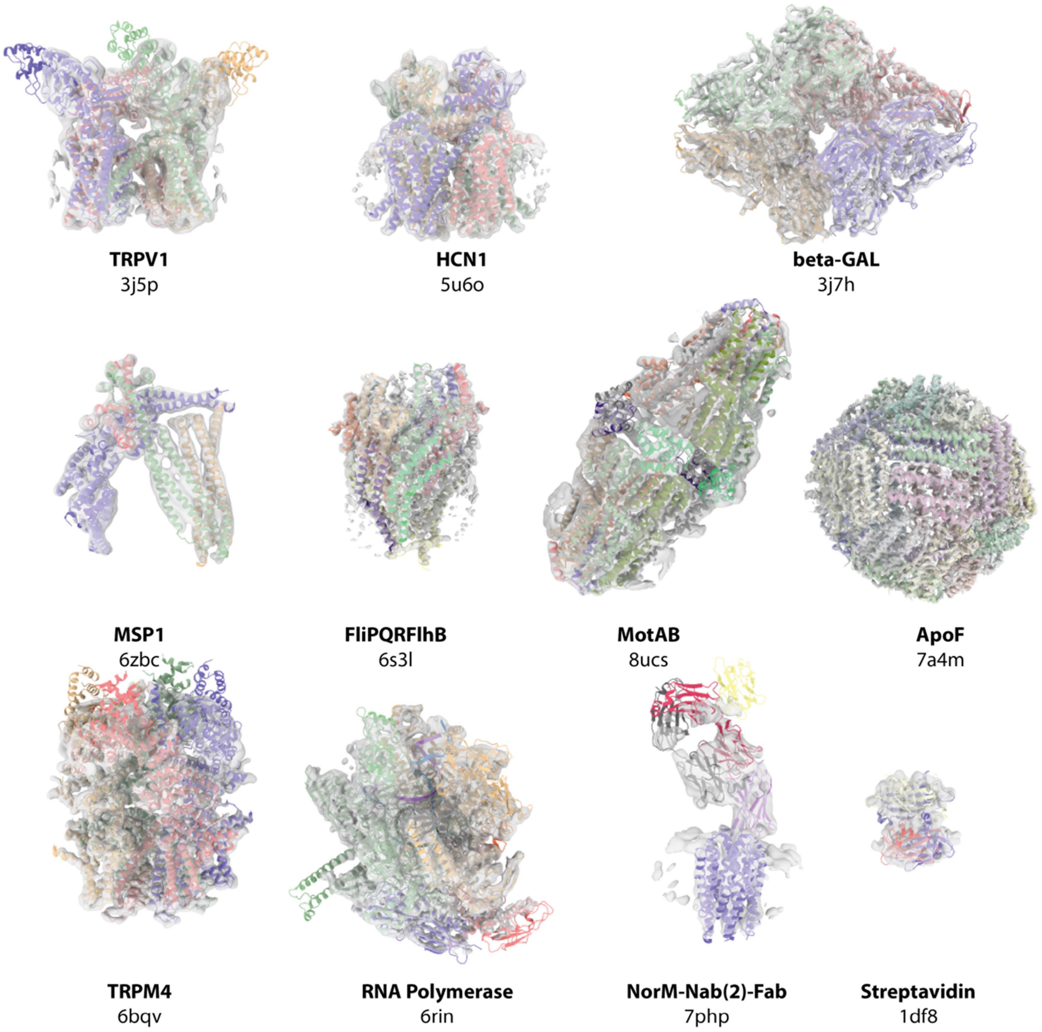

Figure S1 Fitting of atomic coordinates into the medoid *ab initio* volumes

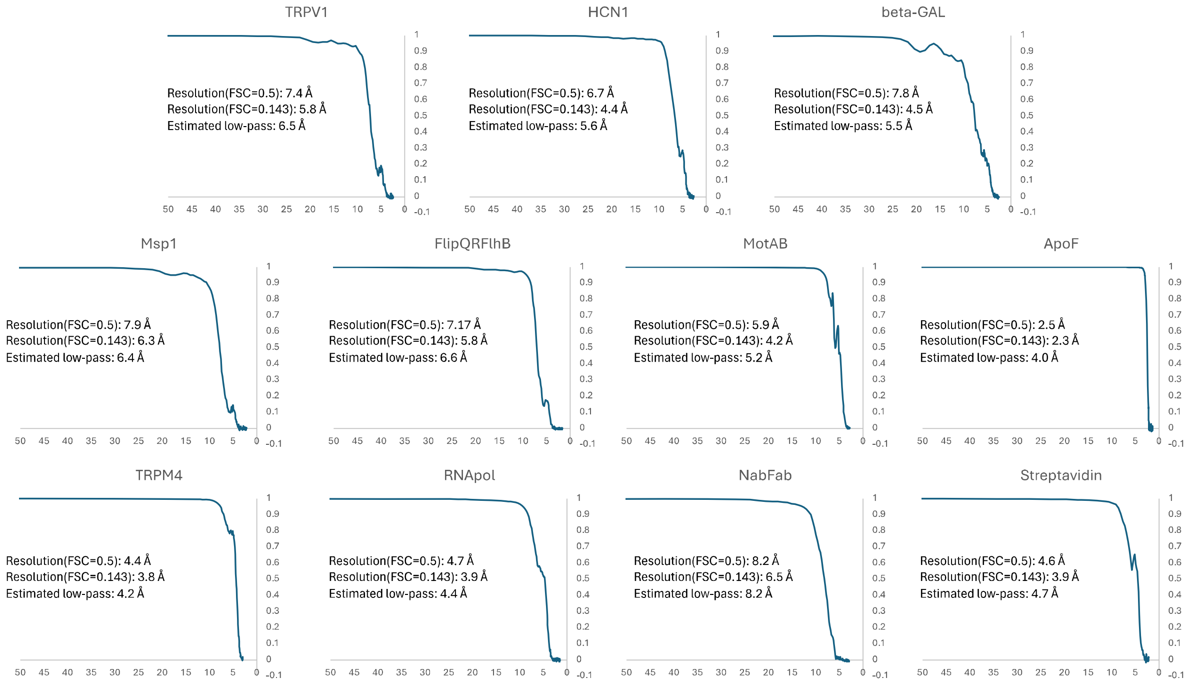

Figure S2 Fourier Shell Correlation (FSC) plots and estimated final low-pass limits

S2. Validation of multi-volume *ab initio* 3D reconstruction on simulated data

We simulated data as previously described by Grigorieff and Lyumkis (Lyumkis *et al.*, 2013) and ran the multi-state *ab initio* 3D reconstruction with het_mode=docked to validate the capability of our algorithm to separate co-existing structural states in the single-particle ensemble.

EMD-1798

Ribosome with elongation factor EF-G bound.

EMD-1799

Ribosome with elongation factor EF-G bound, slight ratcheting of the 30S body and swiveling of the head subunit.

EMD-5030

Ribosome with EF-Tu in place of EF-G, as well as both an A-site and P-site tRNA present.

SNR = 0.1

EMD-1798	0	100	0.5
EMD-1799	0	0	98.7
EMD-5030	100	0	0.8

SNR = 0.05

EMD-1798	0	100	3.5
EMD-1799	0	0	96.1
EMD-5030	100	0	0.4

SNR = 0.02

EMD-1798	0	0.1	99.6
EMD-1799	0	99.7	0.2
EMD-5030	100	0.2	0.2

We concluded that our approach could separate distinct structural states at realistic levels of noise when the nature of the heterogeneity was such that it is meaningful to register the re-projections of the different co-existing structural states to one average volume at the beginning of the search.

References

Lyumkis, D., Brilot, A. F., Theobald, D. L. & Grigorieff, N. (2013). *J Struct Biol* **183**, 377-388.
